# Supplementary figures and images for: The Effect of Background Color on Skin Color Variation of Juvenile Plectropomus leopardus
Source: Animals (Basel). 2022 Nov 29;12(23):3349. doi: 10.3390/ani12233349 (PMC9738834; doi:10.3390/ani12233349)

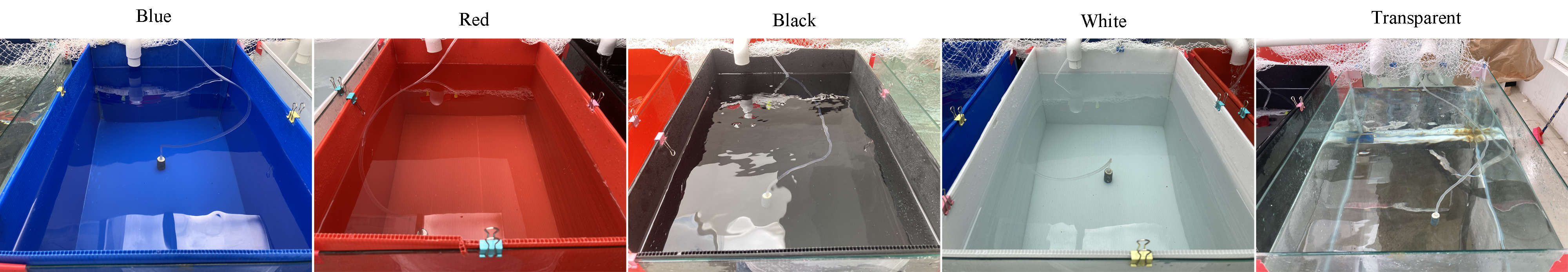

Supplement: Supplementary file 1 [file animals-12-03349-s001.zip › Fig. S1.tif]
